# Dynamics of DNA Methylation in Recent Human and Great Ape Evolution

**DOI:** 10.1371/journal.pgen.1003763

**Published:** 2013-09-05

**Authors:** Irene Hernando-Herraez, Javier Prado-Martinez, Paras Garg, Marcos Fernandez-Callejo, Holger Heyn, Christina Hvilsom, Arcadi Navarro, Manel Esteller, Andrew J. Sharp, Tomas Marques-Bonet

**Affiliations:** 1 Institute of Evolutionary Biology (UPF-CSIC), PRBB, Barcelona, Spain; 2 Department of Genetics and Genomic Sciences, Icahn School of Medicine at Mount Sinai School, New York, New York, United States of America; 3 Cancer Epigenetics and Biology Program (PEBC), Bellvitge Biomedical Research Institute (IDIBELL), L'Hospitalet de Llobregat, Barcelona, Catalonia, Spain; 4 Research and Conservation, Copenhagen Zoo, Frederiksberg, Denmark; 5 Catalan Institution of Research and Advanced Studies (ICREA), Barcelona, Spain; 6 Department of Physiological Sciences II, School of Medicine, University of Barcelona, Barcelona, Catalonia, Spain; University of Chicago, United States of America

## Abstract

DNA methylation is an epigenetic modification involved in regulatory processes such as cell differentiation during development, X-chromosome inactivation, genomic imprinting and susceptibility to complex disease. However, the dynamics of DNA methylation changes between humans and their closest relatives are still poorly understood. We performed a comparative analysis of CpG methylation patterns between 9 humans and 23 primate samples including all species of great apes (chimpanzee, bonobo, gorilla and orangutan) using Illumina Methylation450 bead arrays. Our analysis identified ∼800 genes with significantly altered methylation patterns among the great apes, including ∼170 genes with a methylation pattern unique to human. Some of these are known to be involved in developmental and neurological features, suggesting that epigenetic changes have been frequent during recent human and primate evolution. We identified a significant positive relationship between the rate of coding variation and alterations of methylation at the promoter level, indicative of co-occurrence between evolution of protein sequence and gene regulation. In contrast, and supporting the idea that many phenotypic differences between humans and great apes are not due to amino acid differences, our analysis also identified 184 genes that are perfectly conserved at protein level between human and chimpanzee, yet show significant epigenetic differences between these two species. We conclude that epigenetic alterations are an important force during primate evolution and have been under-explored in evolutionary comparative genomics.

## Author Summary

Differences in protein coding sequences between humans and their closest relatives are too small to account for their phenotypic differences. It has been hypothesized that these differences may be explained by alterations of gene regulation rather than primary genome sequence. DNA methylation is an important epigenetic modification that is involved in many biological processes, but from an evolutionary point of view this modification is still poorly understood. To this end, we performed a comparative analysis of CpG methylation patterns between humans and great apes. Using this approach, we were able to study the dynamics of DNA methylation in recent primate evolution and to identify regions showing species-specific methylation pattern among humans and great apes. We find that genes with alterations of promoter methylation tend to show increased rates of divergence in their protein sequence, and in contrast we also identify many genes with regulatory changes between human and chimpanzee that have perfectly conserved protein sequence. Our study provides the first global view of evolutionary epigenetic changes that have occurred in the genomes of all species of great apes.

## Introduction

The genomic era is characterized by different comparative approaches to understand the effect of genomic changes upon phenotypes. In the context of human evolution, the genomes of all species of great apes have now been sequenced [1]–[4] allowing nucleotide resolution comparisons to understand the evolution of our genome. However, in contrast to these advances in comparative genomic analyses, there has been relatively little progress in the understanding of the evolution of genome regulation [5]–[9].

DNA methylation is an important epigenetic modification found in many taxa. In mammals, it is involved in numerous biological processes such as cell differentiation, X-chromosome inactivation, genomic imprinting and susceptibility to complex diseases [10]–[13]. Promoter hypermethylation is generally thought to act as a durable silencing mechanism [14]. However, the exact relationship between DNA methylation and gene expression is not clear since recent studies have also linked gene body methylation with transcriptional activity and alternative splicing [15]–[17]. At some loci DNA methylation patterns are influenced by the underlying genotype [18]–[20]. However, due to the fact that patterns of DNA methylation can change during development [16], [21], [22] or as a result of environmental factors [23], [24], the exact mechanisms governing DNA methylation states remain unclear.

Most efforts to understand DNA methylation changes in primates have focused on the comparison of human with chimpanzee or macaque [6], [7], [9], [25]. This is largely attributable to the difficulty of obtaining samples from endangered species and the lack of genome sequence for the great apes. The publication last year of draft sequences of the gorilla [2] and bonobo [3] genomes facilitates a more accurate characterization of the species-specific events in all the great ape phylogeny, and interrogation of this epigenetic modification from an evolutionary point of view. Studies to date have found that DNA methylation profiles are, in general, more similar between homologous tissues than between different tissues of the same species [9]. However, differentially expressed genes between human and chimpanzee are often associated with promoter methylation differences, regardless of tissue type, establishing that some differences in the expression rates of genes between the species are associated with differences in DNA methylation. It is estimated that around 12–18% (depending on the tissue) of interspecies differences in gene expression levels could be explained by changes in promoter methylation [9].

Here we present the first comparative analysis of DNA methylation patterns between humans and all great ape species, allowing us to recapitulate the evolution of CpG methylation over the last 15 million years in these species. We used Illumina Methylation450 BeadChips to profile DNA methylation genome-wide in blood-derived DNA from a total of 9 humans and 23 wild-born individuals of different species and sub-species of chimpanzee, bonobo, gorilla and orangutan. We observed that the methylation values recapitulate the known phylogenetic relationships of the species, and we were able to characterize methylation differences that have occurred exclusively in the human lineage and among different great apes species. We also identified a significant positive relationship between the rate of coding variation and alterations of methylation at the promoter level, indicative of co-occurrence between evolution of protein sequence and gene regulation

## Results

### Data filtering

We obtained cytosine methylation profiles of peripheral blood DNA isolated from a set of males and females of nine humans, five chimpanzees, six bonobos, six gorillas and six orangutans (Table S1) using the Illumina HumanMethylation450 DNA Analysis BeadChip assay. Because the probes on the array are designed using the human reference genome, we performed a set of strict filters to remove divergent probes that could bias our methylation measurements. The filtering was based on the number and location of mismatches with their target site in each species genome assembly tested [1]–[4] (Figure S1 and Figure S2, see Methods). This resulted in the retention of 326,535 probes (72%) in chimpanzee, 328,501 probes (73%) in bonobo, 274,084 probes (61%) in gorilla and 197,489 probes (44%) in orangutan, consistent with their evolutionary distance to human. We also applied a second filtering step to remove probes that overlapped with intra-species common variation (see Methods) [26].

Cell heterogeneity may also act as a confounder when measuring DNA methylation, particularly from whole blood [27]. Due to the difficulty of obtaining fresh blood samples from wild-born great apes, we were unable to either isolate a specific blood cell type or measure the cellular composition of the blood samples from which our DNA was extracted. To minimize false positives resulting from different cellular compositions or other confounders, we performed two filtering steps. First, we removed CpG sites that showed differential methylation in human between whole blood and each of the two most abundant subtypes of blood cell (CD4+ T-cells and CD16+ neutrophils, see Methods). Second, we required a minimum threshold of at least 10% change in mean methylation (mean β-value difference ≥0.1) at each CpG in order to define differential methylation between species. As a result of this threshold, differences in other cell types that account for <10% of the cellular composition of blood, are unlikely to affect our results (see Methods).

In this work we used two different datasets: i) we confined our analysis to 114,739 autosomal probes and 3,680 probes on the X chromosome that were directly comparable across all the species to facilitate an unbiased comparison of human and all great apes (32 individuals), and ii) we used 291,553 shared autosomal probes between humans and chimpanzees to compare these two species. We performed separate analyses of autosomal and sex-linked probes to prevent confounding effects of X chromosome inactivation on DNA methylation between males and females [13]. Unless specifically mentioned, all results presented below refer to analysis of autosomal probes only.

### Phylogenetic relationships

**Figure 1:**
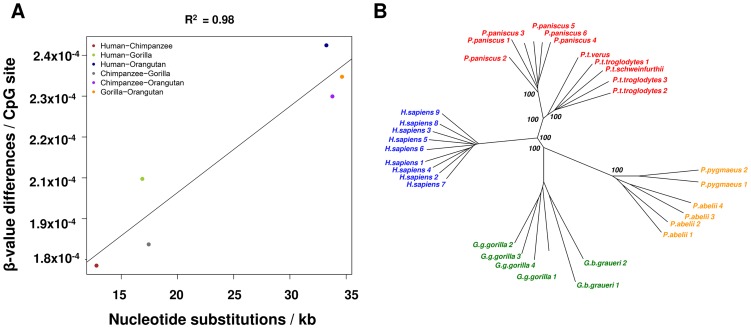
Methylation patterns mimic sequence based phylogenetic relationships. (A) Methylation changes correlate with DNA sequence changes. x-axis shows the number of nucleotide substitutions between two species per kb, y-axis shows the changes in methylation based on β-values. (B) Neighbor-joining tree based on methylation data from probes with a perfect match in all reference genomes (31,853 autosomal CpGs). Bootstrap values (1,000 permutations) are shown for each node.

To investigate the global correspondence of DNA sequence differences between species and the degree of methylation changes, we examined the Enredo-Pecan-Orthus (EPO) whole-genome multiple alignments of human, chimpanzee, gorilla, and orangutan [Ensemble Compara.6_primates_EPO] [28], [29] and we calculated pairwise distances between these four species. Upon comparison of these sequence distances and methylation data (see Methods), we observed a high global correlation between sequence substitution and methylation divergence (R^2^ = 0.98, p = 0.0003) (Figure 1A). We then constructed a neighbor-joining phylogenetic tree based on the methylation levels of the 114,739 autosomal CpGs measured in all individuals and species (Figure S3). This tree accurately recapitulates the known evolutionary relationships of great apes, including the separation at sub-species level of the *Pan*, *Gorilla* and *Pongo* genera. These results are also maintained when using only the subset of probes that have a perfect match (n = 31,853) to each of the primate reference genome and contain no common polymorphisms suggesting that that methylation levels are associated with the evolutionary history of these species (Figure 1B).

### Lineage-specific methylation changes

Due to the relatively recent origin of all partitions within genera of great apes [2]–[4] and our sample size, we focused our analysis on changes at the genus taxonomic level. To identify only those methylation differences that represent fixed changes between these groups and to avoid possible artifacts due to intraspecific polymorphism, we retained only those CpGs with low methylation variance within each genus (intragenus standard deviation <0.1). This filtering step resulted in the removal of 1,377 CpGs in human, 5,224 in the *Pan* sp., 5,289 in *Gorilla* sp. and 5,740 in *Pongo* sp., with the resulting final set being 99,919 CpGs shared across all five species, covering 12,593 genes (≥2 probes within a 1 kb interval and overlapped with RefSeq genes, −1500 bp transcription start site (TSS) to 3′UTR). The proportion of sites removed in this step are consistent with the relative population diversity within each of these species [2]–[4], [30].

**Figure 2:**
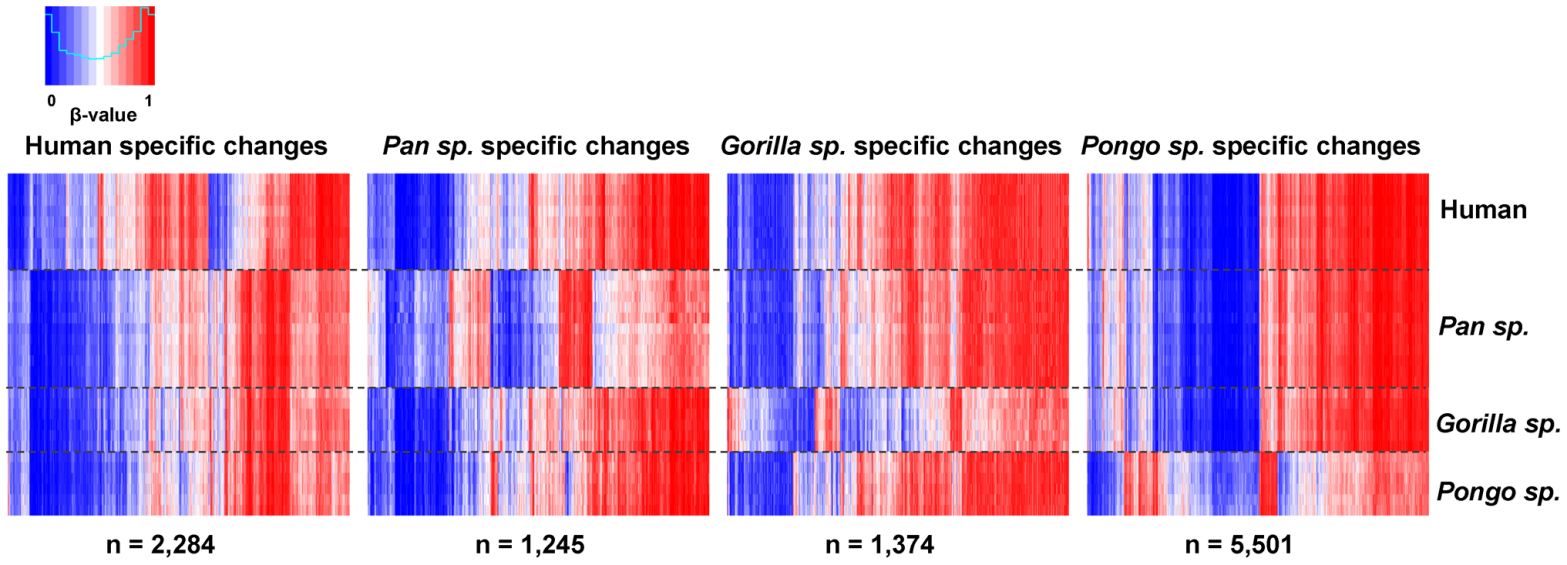
Differentially methylated CpG sites in each great ape genus. Heat maps showing genus specific differentially methylated CpG sites. We found 2,284 human-specific differentially methylated CpGs, 1,245 specific to *Pan* species, 1,374 specific to *Gorilla* species and 5,501 changes specific to *Pongo* species. Each vertical line represents a single CpG, with each row showing the β-value obtained in each individual tested.

Approximately 22% of the sites tested (n = 21,884 CpGs) showed no significant changes among any of the species (conserved sites: Wilcoxon rank-sum test, FDR-adjusted p>0.05 and mean β-value difference all cases <0.1). Comparison of genes linked with these sites showed an enrichment of Gene Ontology (GO) categories for fundamental cellular processes. In contrast, we identified 2,284 human-specific (2.3%) differentially methylated CpGs, 1,245 specific to *Pan* species (1.2%), 1,374 specific to *Gorilla* species (1.4%). and 5,501 changes specific to *Pongo* species (5.5%) (Wilcoxon rank-sum test, FDR-adjusted p<0.05 and mean β-value difference ≥0.1, see Methods) (Figure 2 and Table S2). We clustered these sites into regions with at least two nearby differentially methylated CpGs (<1 kb interval) and overlapped with RefSeq genes (−1500 bp from TSS to 3′UTR). Doing this, we identified 171 genes that show human specific methylation patterns, 101 genes in *Pan* species, 101 genes in *Gorilla* species and 445 genes in *Pongo* species (Table S3). We observed that this spatial aggregation of differentially methylated sites is significantly non-random (random permutation compared to all 99,919 CpGs used in our analysis, p<0.0001, see Methods) and a simple Likelihood Ratio Test also suggested a non-homogenous rate of methylation changes in the human and great ape evolution (LRT, p<10^−5^, see Text S1).

Using the Genomic Regions Enrichment of Annotation Tool (GREAT) [31] (see Methods) we identified significant enrichments (FDR-corrected p<0.05) for several biological processes associated with lineage-specific differentially methylated genes. Within the human-specific differentially methylated regions most of the categories found were related with the circulatory system, as expected from testing blood-derived DNA. However, we also found enrichment for terms related to development and neurological functions, including semicircular canal formation and facial nucleus development (Table S4). The use of disease ontology terms showed that mutations in several of these genes are known to be associated with diseases including Möbius syndrome, Asperger's syndrome and malignant hyperthermia. In the *Pan* genus (chimpanzee and bonobo) we observed significant enrichments among genes involved in epithelial development and the respiratory system, while in *Pongo* species (orangutan) enriched categories included a variety of basic metabolic and reproductive processes (Table S4).

**Figure 3:**
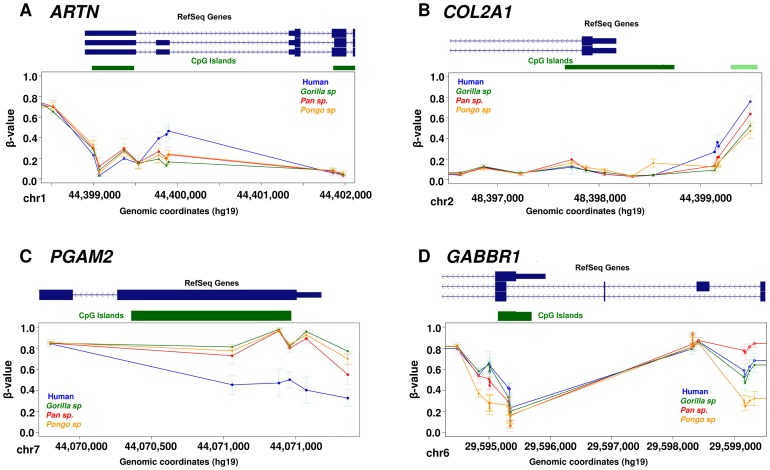
Examples of differentially methylated genes. Each data point represents the mean β-value of the group and whiskers show 2 standard deviations above and below the mean. (A) *ARTN* is a neurotrophic factor and it shows hypermethylation of 3 CpG sites associated with the long isoform specifically in human. (B) *COL2A1* shows hypermethylation of 4 CpG sites at the promoter specifically in human. This gene encodes the alpha-1 chain of type II collagen, which is found primarily in cartilage, the inner ear and the vitreous humor of the eye. (C) *PGAM2* shows hypomethylation of CpG sites at the promoter specifically in human. *PGAM2* is an enzyme involved in the glycolitic pathway, mutations in which are associated with muscle cramping and intolerance for strenuous exercise. (D) *GABBR1* shows a complex methylation pattern in which human and gorilla shows similar pattern of methylation, orangutan shows relative hypomethylation, while chimpanzees and bonobos show increased methylation levels at TSS and intermediate levels associated with the short isoform of this gene. *GABBR1* is a neuronal receptor involved in synaptic inhibition, slow wave sleep, muscle relaxation and sensitivity to pain.

We found a particular set of genes with methylation changes specifically in the human lineage including examples such as *ARTN*, *COL2A1* and *PGAM2* (Figure 3). *ARTN* is a neurotrophic factor which supports the survival of sympathetic peripheral neurons and dopaminergic neurons. *COL2A1* encodes the alpha-1 chain of type II collagen, which is found primarily in the cartilage, the inner ear and the vitreous humor of the eye. Mutations in this gene are associated with several developmental syndromes [32]. *PGAM2* is an enzyme involved in the glycolytic pathway, mutations in which are associated with glycogen storage disease [MIM: 261670], a defect that causes muscle cramping, myoglobinuria and intolerance for strenuous exercise. In addition to the identification of regions showing changes in a single species, we also detected loci with more complex changes in methylation profiles among great apes. One example is the promoter region associated with different isoforms of the *GABBR1* gene (Figure 3D). This gene encodes the GABA_B_ receptor 1, a G protein-coupled receptor involved in synaptic inhibition, hippocampal long-term potentiation, slow wave sleep, muscle relaxation and sensitivity to pain. While human and gorilla have *GABBR1* promoter methylation patterns that are broadly similar to each other, orangutan shows relative hypomethylation across this region. In contrast chimpanzee and bonobo show increased methylation specifically at the TSS of long *GABBR1* isoforms, and intermediate methylation levels associated with the short isoform. These data suggest some epigenetic differences among primates are associated with isoform regulation.

### Functional context of differentially methylated sites in the genome

**Figure 4:**
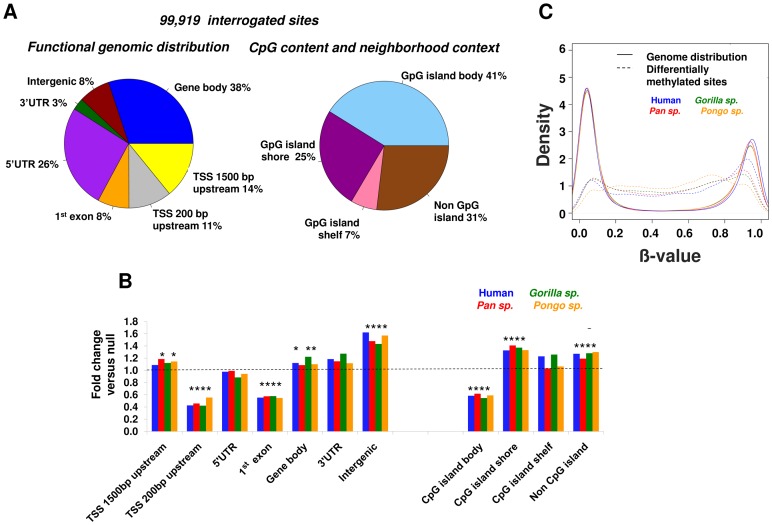
Location of differential methylation in primate genomes. (A) Distribution of 99,191 CpG sites interrogated in all great ape species. Left: Gene-centric functional distribution of methylation changes : 1,500 bp upstream of gene TSSs, 200 bp upstream of TSSs, 5′UTR, 1^st^ exon, gene body, 3′ UTR and intergenic. Right: CpG-island centric distribution: CpG island, shore (±2 kb flanking CpG islands), shelf (2–4 kb from CpG islands). (B) A non-random distribution of methylation changes in recent primate evolution. We observe an excess of differential CpG methylation within the first 1,500 bp upstream of gene TSSs, gene bodies, intergenic regions, shore regions flanking CpG islands and non-CpG island regions. In contrast DNA methylation tends to be relatively conserved close to gene transcription start sites (−200 bp of TSS to 1^st^ exon), and in the body of CpG islands. Each bar shows the relative enrichment for differential methylation versus that expected under a null distribution. * denotes p<0.0001 (permutation test). (C) Density plot showing the distribution of methylation levels of differentially methylated sites compared to that in the rest of the genome. Sites of evolutionary change among great apes have a significantly different distribution (p = 2.2×10^−16^ Kolmogorov-Smirnov test).

We observed a highly non-random distribution of the differentially methylated CpGs (Figure 4A and 4B) in relationship to gene annotations and CpG density. From the functional distribution standpoint, there was a significant excess of changes (p<0.0001, permutation test, see Methods) for sites located within 1,500 bp upstream of gene TSSs, gene bodies and intergenic regions, and from the CpG content standpoint, differential methylation occurred preferentially in CpG shores (±2 kb CpG island) and non-CpG island regions. These results highlight CpG shores as epigenetically variable regions, as it has been observed in human development and disease [12], [33]. In contrast, the regions immediately surrounding gene TSSs (−200 bp of the TSS and 1^st^ exon) and CpG islands showed relative conservation of methylation.

We also observed a significant difference in the distribution of methylation levels at differentially methylated sites compared to the rest of the genome (Figure 4C). While the overall genome-wide pattern of methylation levels shows a strongly bi-modal distribution, with most sites having either very high or very low methylation levels, sites of evolutionary change have a significantly different distribution to genome wide distribution (p = 2.2×10^−16^ Kolmogorov-Smirnov test), showing intermediate methylation levels, which has been shown to be a hallmark of distal regulatory elements. [34].

### X-chromosome inactivation

In female mammals, X chromosome inactivation (XCI) is maintained via a number of epigenetic marks, including altered DNA methylation [35], [36]. Consistent with a role in XCI, the majority of sites we identified on the X chromosome in great apes showed relatively higher methylation levels in females versus males due to the contribution from the inactive X chromosome (63%, p = 0.005, Figure S4A). We searched for CpG sites on the X chromosome presenting no significant changes between males and females in a specific lineage (mean β-value difference <0.1) but showing significant gender differences in all the other species (see Methods). This analysis identified 22 CpGs in human, 59 in chimpanzee and bonobo, 72 in gorilla and 41 in orangutan (Table S5). Some regions are particularly interesting such as the *MID1* gene which has been previously reported as a gene subject to X-inactivation in humans but not in mouse [37]. Our results suggest that this gene may escape XCI in the *Pan* lineage, but not in all other great apes. Similarly the *HTR2C* gene shows multiple probes upstream of the TSS with similar patterns of methylation in both male and female humans, potentially suggesting that this gene escapes XCI in the human lineage. In contrast, the same sites show significantly higher methylation levels in females compared to males in all other primate species, suggesting that in these species *HTR2C* may be subject to XCI (Figure S4B). Using published RNAseq data [38], we did not observe a female-specific increase in *HTR2C* gene expression for in humans, although we note that many genes escaping XCI show no clear sex differences in expression levels [39].

### Pairwise comparison of human and chimpanzee

To maximize the identification of altered methylation patterns between human and our closest living relative, the chimpanzee, we performed a pairwise comparison of these two species using a larger dataset of 289,007 filtered probes common to human and chimpanzee. We used the chimpanzee species and not the whole genera to make use of the better annotation in the genome reference assembly for this species compared to the rest of non-human primate genome reference assemblies [1]. We identified 16,365 sites that showed significant hypermethylation in human, and 9,693 sites showing significant hypomethylation (FDR-adjusted p<0.05, β-value difference ≥0.1). This represents ∼9% of the total number of sites tested, and includes ∼2,500 genes (≥2 differentially methylated CpGs within a 1 kb interval and overlapped with RefSeq genes, −1500 bp TSS to 3′UTR).

**Figure 5:**
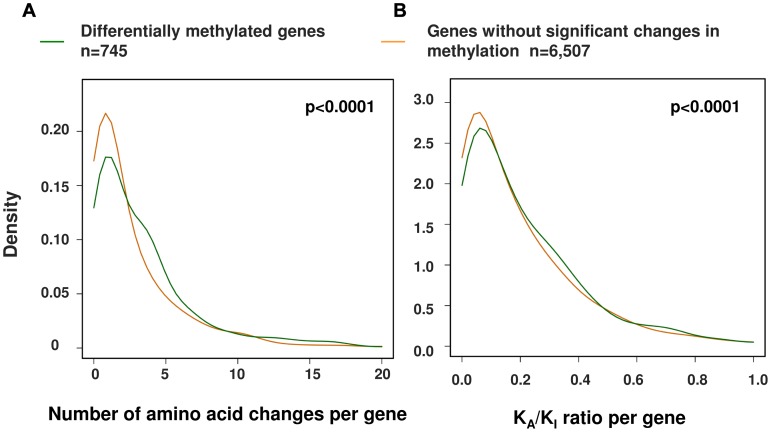
A significant relationship between changes in promoter methylation and protein evolution between human and chimpanzee. We performed a comparison of alterations in promoter methylation with (A) the frequency of amino-acid alterations and (B) the relative rate of coding to non-coding variation with genes (K_A_/K_I_) between human and chimpanzee. Using both metrics we observed a significant association between the rate of protein evolution and epigenetic regulatory changes. P-values are based on 1,000 permutations (Differentially methylated genes, n = 745; genes without significant changes in methylation, n = 6,507).

**Figure 6:**
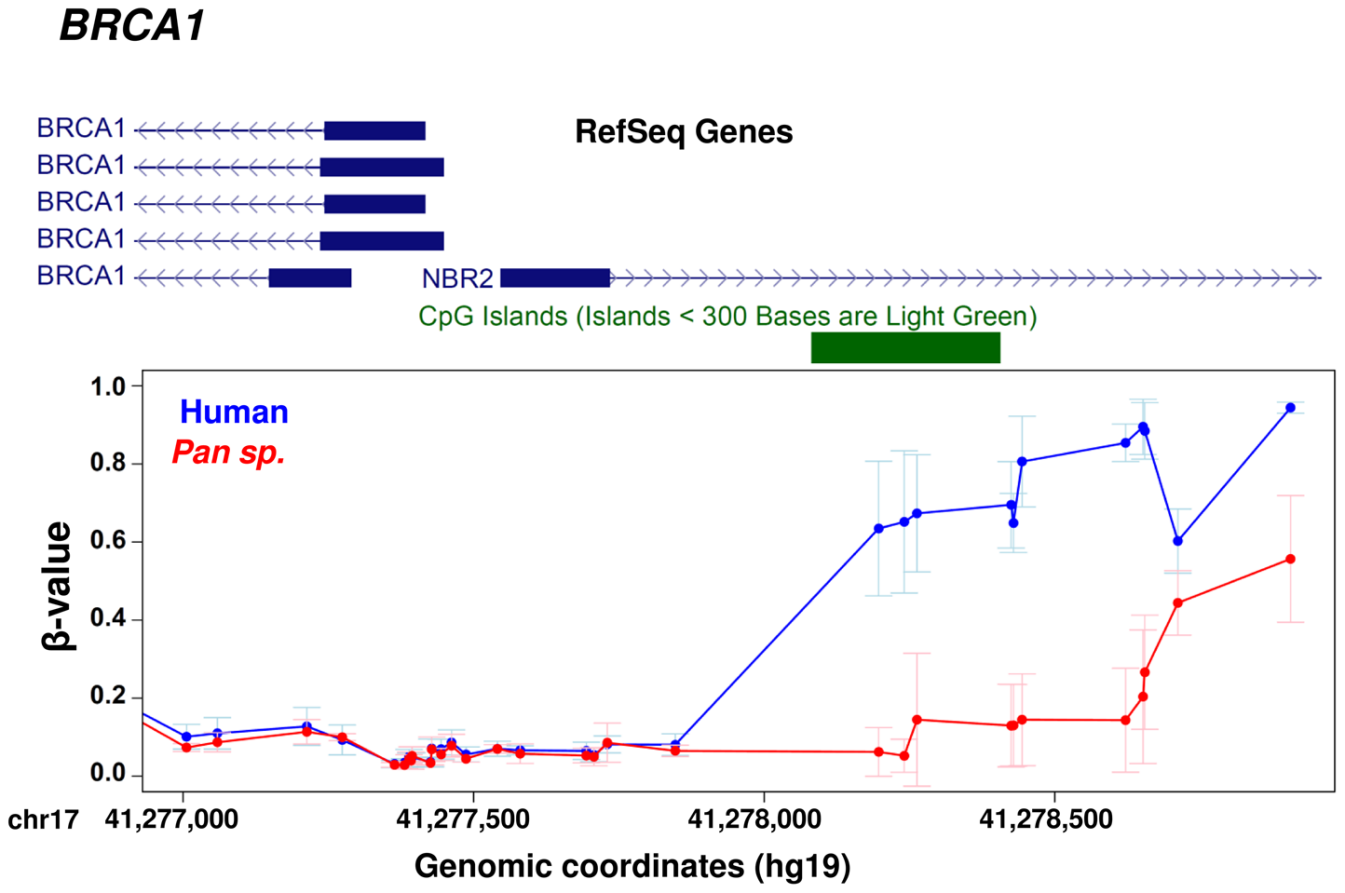
Example gene showing methylation differences between human and chimpanzee at promoter level. *BRCA1* provides an example of co-occurrence between protein sequence evolution and gene regulation. The *BRCA1* gene shows changes in DNA methylation in a regulatory region upstream of the TSS [40] and a K_A_/K_I_ ratio of 0.69 between human and chimpanzee.

Using this larger dataset, we then investigated the relationship between the evolution of protein coding sequences and epigenetic change at promoter level. Using a curated set of 7,252 human∶chimpanzee 1∶1 orthologs [1] we identified 745 genes (∼10% of those tested) that showed at least two differentially methylated sites at the promoter (−1500 bp from the TSS to 1^st^ exon, see Methods). We then compared both the number of amino acid changes and the K_A_/K_I_ ratio (the number of coding base substitutions that result in amino acid changes as a fraction of the local intergenic/intronic substitution rate) of these differentially methylated genes against the remainder [1] (Figure 5). We observed a significant difference in both the number and rate of non-synonymous amino acid changes between genes with altered promoter methylation compared to those without significant methylation differences (p<0.0001, permutation test) suggesting that rapid evolution at the protein coding level is frequently coupled with epigenetic changes in the promoter. We also observed similar results when using only those probes with a perfect match to the chimpanzee reference genome (Figure S5). An interesting example is the *BRCA1* gene, which contains 32 amino acid changes between human and chimpanzee and has a K_A_/K_I_ ratio of 0.69 (three times the average of all orthologous genes). This gene shows large differences in methylation ∼1–1.5 kb upstream of the TSS (Figure 6). Previous studies have shown that methylation changes of this same region are associated with altered *BRCA1* expression [40].

In contrast, we also observed 184 genes that show perfect human:chimpanzee conservation at the amino acid level, yet they show significant epigenetic differences at their promoter (Table S6). Within this set of genes, we observed significant enrichments for categories related with gene expression (table S7) [41], [42].

**Figure 7:**
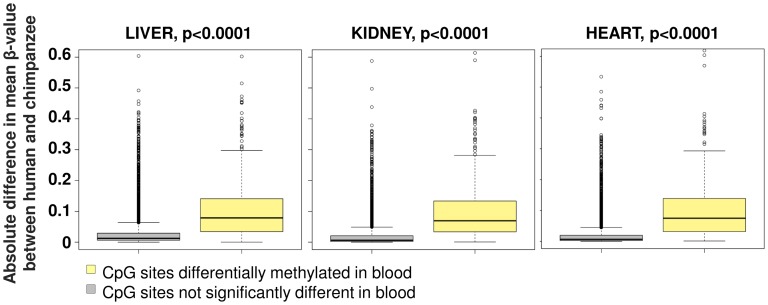
Conservation of human-chimpanzee differentially methylated sites among multiple somatic tissues. Differentially methylated sites we identified using whole blood of human and chimpanzee were compared to a previous study that used the Illumina HumanMethylation27 DNA Analysis BeadChip to study liver, kidney and heart tissue in an independent population of humans and chimpanzees [19]. We found a significant trend for sites that are differentially methylated in blood to also show higher human-chimpanzee divergence in these other tissues (yellow box plot, n = 457) suggesting a conservation across other somatic tissues compared to non-differentially methylated sites in blood (grey box plot, n = 7,942).

As our survey of evolutionary changes in primate DNA methylation patterns utilized DNA derived from whole blood, we tested whether these changes are also present in other somatic tissues by comparing against an independent dataset. A previous study [9] utilized a similar array platform, although with a much reduced probe density, to compare DNA methylation levels in humans and chimpanzees using DNA isolated from heart, liver and kidney. Comparing the 457 sites common to both datasets that we defined as differentially methylated in blood samples versus these three other tissues, we observed a highly significant trend for methylation differences identified between human and chimpanzee to be conserved across all four tissue types (Figure 7).

## Discussion

The primary focus to date for understanding human evolution from a comparative genomic perspective has been the study of changes in DNA sequence and gene expression levels [43]–[45]. Our study of DNA methylation profiles among human and great apes adds to this wealth of information, reinforcing the view that epigenetic changes contribute significantly to species divergence, and therefore they should be considered in studies of human evolution.

In this study, one of the main challenges was the technical limitation stemming from the use of arrays designed against the human genome to profile methylation patterns in great ape species with divergent genomes. We utilized a set of filters to account for these differences, and were also able to replicate the results even after limiting our analysis to those probes with 100% identity in each of the non-human reference genome assemblies. Supporting a biological role for our findings, we observed that the clustering of differential methylation within each species was highly non-random, and showed significant enrichments within functional genomic elements.

From a biological perspective, it is conceivable that differences in the constitutive fractions of whole blood between species might introduce a bias due to the fact that different cell types possess distinct epigenomes [27]. This limitation is shared by nearly all comparative molecular studies of primary tissues from endangered species (i.e. great apes) due to the difficulty of obtaining relevant samples, especially in the case of wild-born individuals as the ones used in this study. However, in order to minimize this problem we removed all CpG sites that vary significantly between whole blood and the most abundant cell populations in blood. We further required a minimum threshold of 10% change in global methylation between sites in these species in order to identify differentially methylated sites, meaning that changes in the prevalence of minor cell fractions would not influence the results. Finally, while all samples were obtained from adult individuals, we could not match the ages perfectly among all samples, so in order to compensate for this effect, and to minimize the effects of intraspecific polymorphism, we focused our study on sites with low intragenus variance.

Our results show that ∼9% of the CpGs we assayed showed significant methylation differences between human and chimpanzee, including the promoter regions of 745 genes (10% of those tested). We estimate that over 2,500 genes present at least some methylation changes between human and chimpanzees (≥2 differentially methylated sites separated by ≤1 kb), a higher number than that known to be affected by copy number variation or under positive selection in the same species [46]–[49]. Although the arrays we used do not provide a complete and unbiased coverage of the genome, these data suggest that epigenetic changes have been frequent during recent primate evolution and represent an important substrate for adaptive modification of genome function. Underlining this idea, the changes we observed among primates are highly enriched for sites showing intermediate DNA methylation levels. Previous studies have shown that such methylation values are often a hallmark of distal regulatory elements [34], suggesting that many epigenetic changes occurring among human and great ape species impact transcriptional regulation. Consistent with these findings, we detected global enrichments for epigenetic change within known regulatory regions, including distal regions upstream of gene transcription start sites and regions flanking CpG islands (termed ‘CpG shores’).

We observed that the great ape phylogeny can be recapitulated from methylation data alone. Potential explanations for this are that methylation values could be driven by proximal DNA changes that were not controlled in this study, or that epigenetic changes have occurred independently of DNA sequence but are subject to similar rates of change either through selective pressures or neutral drift.

Interestingly, we also identified a significant positive relationship between the rate of coding variation within genes and alterations of promoter methylation, suggesting a co-occurrence between changes in protein sequence and gene regulation that may be related to expression changes in fast evolving genes [50]. In contrast, and consistent with previous analysis indicating the importance of regulatory changes in evolution [51], our study also identified scores of genes that are perfectly conserved at the amino acid level between human and chimpanzee, yet showing significant epigenetic change between these two species. Furthermore, gene ontology analysis of this set showed that they are significantly enriched for the functional category of gene expression. These observations highlight the evolutionary importance of epigenetic changes that affect gene regulation, and also demonstrate that sequence-based studies are insufficient to capture the full spectrum of evolutionary change.

Overall our analysis identified >800 genes with significantly altered methylation patterns specifically within each species of human and great apes, including 171 with a methylation pattern unique to humans. Analysis of these 171 genes identified interesting enrichments for a number of functional categories that could suggest a relationship to human-specific traits. For example, we observed that genes involved in the regulation of blood pressure and development of the semicircular canal of the inner ear among others, were all highly enriched for DNA methylation changes specifically in the human lineage. While major changes in circulatory physiology are required for upright locomotion, the inner ear provides sensory input crucial for maintaining balance. Furthermore, a previous study of primates and other mammals has shown that the size of the semicircular canals is correlated with locomotion and with relatively larger canals found in species that utilize fast or agile movement [52]. While these trends hint at the potential importance of epigenetic changes in the evolution of several human-specific features, we caution that at this stage they should be considered as preliminary, as our studies were performed using DNA derived from whole blood, and it is well known that epigenetic patterns often vary widely between different tissues of an organism [27]. Therefore further studies in physiologically relevant tissues will be required to confirm the significance of these findings. However, we note that comparison with previously published data [9] suggests that many of the changes in DNA methylation that we detected between blood of human and chimpanzee appear to be conserved across several other tissues, suggesting that inter-specific differences observed in blood can in some cases be informative for other tissues.

Although sequencing studies have undoubtedly provided major advances in our understanding of primate evolution, our analysis of primate epigenomes unveils many novel differences among the great apes that are not apparent from purely sequence-based approaches. Of particular note is the fact that we identify enrichments in multiple independent functional gene categories which suggests that regulatory changes may have played a key role in the acquisition of human-specific trait. Therefore, epigenetic alterations likely represent an important facet of evolutionary change in primate genomes. Future studies that integrate epigenetic data with recent detailed maps of functional elements, selective constraint and chromatin interactions in the human genome [53]–[55] will likely provide many novel insights into genomic and phenotypic evolution.

## Methods

### Ethics statement

The non-human research has been approved by the ethical committee of the European Research Union. No living animal has been used and DNA has been obtained during standard veterinary checks. Methylation profiling of human subjects was approved by the Institutional Review Board of the Icahn School of Medicine at Mount Sinai (HS#: 12-00567 HG).

### Hybridization and normalization

We obtained methylation data from peripheral blood DNA extracted from 9 adult humans, 5 chimpanzees, 6 bonobos, 6 gorillas and 6 orangutans. All individuals were unrelated adults and the non-human primates were all wild born. DNA samples were bisulfite converted, whole-genome amplified, enzymatically fragmented, and hybridized to the Infinium HumanMethylation450 BeadChip which provides quantitative estimates of methylation levels at 482,421 CpG sites distributed genome-wide. The assay was performed according to the manufacturer's instructions. The BeadChip array data discussed in this publication have been deposited in NCBI's Gene Expression Omnibus and are accessible through GEO Series accession number GSE41782. Due to the low density of probes targeting non-CpG dinucleotides (<0.7%) on the array, we focused our study on CpG sites.

Since the 50 bp probes on the array were designed against the human reference genome but we performed hybridizations utilizing DNA from different great ape species, we first mapped the probe sequences to the chimpanzee (panTro3), bonobo (panPan1), gorilla (gorGor3) and orangutan (ponAbe2) reference genomes using BWA [56], allowing a maximum edit distance of 3. We then assessed probe performance as a function of the number and relative location of sequence differences at the probe binding site in each primate genome (Figure S1 and Figure S2). Based on this analysis, in each species we only retained those probes that had either a perfect match, or had 1 or 2 mismatches in the first 45 bp but no mismatches in the 3′ 5 bp closest to the CpG site being assayed. We also removed all probes that contained human SNPs with minor allele frequency ≥0.05 within the last 5 bp of their binding site closest to the CpG being assayed [57]. Using published SNP data [26] for each species we removed probes containing SNPs with minor allele frequency ≥0.15 within the last 5 bp of their binding site closest to the CpG being assayed. We also removed all probes that contained more than two SNPs with minor allele frequency ≥0.15 in the first 45 bp.

Methylation values for CpG sites in each sample were obtained as β-values, calculated as the ratio of the methylated signal intensity to the sum of both methylated and unmethylated signals after background subtraction (β-values range from 0 to 1, corresponding to completely unmethylated and fully methylated sites, respectively). Within each individual, probes with a detection p>0.01 were excluded. We performed a two color channel signal adjustment and quantile normalization on the pooled signals from both channels and recalculation of average β-values as implemented in “lumi” package of R [58]. The Illumina Infinium HumanMethylation450 BeadChip contains two assay types (Infinium type I and type II probes) which utilize different probe designs. As the data produced by these two assay types shows distinct profiles (Figure S6), to correct this problem we performed a BMIQ (beta mixture quantile method) [59] on the quantile normalized data sets.

Using a published human data set [27] we identified differentially methylated sites between whole blood and CD4+ T-cells, and between whole blood and CD16+ neutrophils, representing the two most abundant cell fractions of blood (comprising ∼13% and ∼65%, respectively) (Wilcoxon rank-sum test, FDR-adjusted p<0.05 and mean β-value difference in each case ≥0.1). These sites (n = 10,151) were removed to mitigate potential confounders due to differing proportions of blood cell types among primates, leaving for comparison only those sites that do not significantly vary among the most abundant cell types of blood. β-values can be interpreted as the percentage of methylation at a given site. A β-value of 0.1 indicates that there has been a change in methylation in 10% of the molecules tested. Because our analyses required a mean β-value difference >0.1 to achieve significance, this threshold means that changes in blood cell fractions representing <10% of whole blood will be unlikely to affect our results. The final dataset after all filtering steps comprised 114,739 probes shared across all great ape species, and 291,553 probes shared between human and chimpanzee.

### Phylogenetic relationships

To investigate the global correspondence of DNA sequence differences between species and the degree of methylation changes, we examined the Enredo-Pecan-Orthus (EPO) whole-genome multiple alignments of human, chimpanzee, gorilla, and orangutan [Ensemble Compara.6_primates_EPO] [28], [29]. Considering only those blocks with alignments for all great apes, we first excluded regions containing gaps or indels and then calculated pairwise distances between these four species based on the frequency of single nucleotide substitutions. To calculate the global changes in methylation we used a distance matrix, we first averaged the β-values per probe within a species and then calculated the difference between two species using Euclidean distances.

We built phylogenetic trees based on the methylation states of 114,739 filtered probes (perfect match probes and probes containing 1 or 2 mismatches in the first 45 bp) (Figure S3). We used the “ape” R package to construct the phylogenetic tree using the Neighbor-Joining algorithm and 1,000 bootstraps of the resulting tree [60]. We repeated the analysis using only the subset of probes with a perfect match to each of the primate reference genome assemblies (n = 31,853) (Figure 1B).

### Differentially methylated sites

To identify only those methylation differences that represent fixed changes between genera, we retained only those CpGs with low methylation variance within each genus (intragenus standard deviation <0.1). This filtering step resulted in the removal of 1,377 CpGs in human, 5,224 in the *Pan* genus, 5,289 in *Gorilla* and 5,740 in *Pongo*, with the resulting final set being 99,919 CpGs shared across all five species.

We performed six pairwise comparisons among groups (Human-*Pan* species/Human-*Gorilla* species/Human-*Pongo* species/*Pan* species-*Gorilla* species/*Pan* species-*Pongo* species/*Gorilla* species-*Pongo* species). We defined a site to be genus-specific differentially methylated if all three comparisons with other groups were significant (Wilcoxon rank-sum test, FDR-adjusted p<0.05) and mean β-value difference in each case ≥0.1. We also tried other statistical approaches (linear modeling, limma package, [59]) and obtained very similar results (concordance for 98% of the sites).

All coordinates quoted are based on hg19. We intersected human probe coordinates provided by Illumina with RefSeq genes, retaining CpG sites overlapping genes (−1500 bp from TSS to 3′UTR). We defined a gene to be differentially methylated if there were at least two differentially methylated CpG sites separated by ≤1 kb. To assess significance of these observations we performed a permutation test, as follows. Based on the number of differentially methylated sites detected in each species (Human = 2,284; Pan = 1,245; Gorilla = 1,374; Orangutan = 5,501) we randomly sampled from the 99,919 CpGs and then determined the number of clusters (at least two differentially methylated CpG sites separated by ≤1 kb), repeating this process 10,000 times to create the null distribution. The p-value corresponded to the number of times that differentially methylated clusters appeared within the null distribution divided by the number of permutations (n = 10,000).

The Genomic Regions Enrichment of Annotations Tool (GREAT version 2.0.1) [31] was utilized to identify significant enrichments (FDR-corrected p<0.05) for Gene Ontology biological processes. While tools for identifying enriched GO terms are usually based on genes, GREAT permits the assignment of biological function to non-coding genomic regions by analyzing the annotations of nearby genes. For this analysis regulatory regions were associated to the single nearest gene situated within 10 kb. The background data set was the 99,919 CpG sites interrogated in all great ape species. In order to evaluate the positional context of the differentially methylated sites, we compared the distribution of these 10,404 sites detected among the primate species with all 99,919 CpGs tested. Permutation p-values were calculated as described above using 10,000 iterations.

### X-chromosome inactivation

We performed two color channel signal adjustment and quantile normalization on males and females separately. Due to the different methylation pattern in females no BMIQ normalization was done in this data set. For studies of DNA methylation on the X-chromosome that might be linked with XCI between species, we searched for CpG sites presenting no significant changes between males and females in a specific lineage (mean β-value difference <0.1) but showing significant changes in all the other species (mean β-value difference between sexes >0.1).

### Human-chimpanzee analysis

The number of probes shared between human and chimpanzee after applying our mapping and SNP filters was 291,554. Based on this set of probes, we performed a separate two color channel signal adjustment and quantile normalization of the raw data using only human and chimpanzee samples. We performed a BMIQ normalization to correct the probe design bias. After excluding probes with a standard deviation within either species >0.1 we retained a total of 289,007 probes. Differentially methylated sites were those with p<0.05 (Wilcoxon rank-sum test, FDR-adjusted p<0.05) and a mean β-value difference ≥0.1.

From the total set of 13,454 human:chimpanzee orthologous genes [1], we removed genes with <150 or >1500 amino acids, and then compared the number of amino acid changes and the K_A_/K_I_ ratio of genes with robust alterations of promoter methylation (mean β-value difference of top 2 probes within promoter ≥0.1, considering CpGs located ≤1,500 bp upstream of Refseq gene TSSs, in the 5′UTR or the 1^st^ exon, n = 745) versus those without methylation changes (n = 6,507). The Gene Ontology enRIchment anaLysis and visuaLizAtion tool (GOrilla) [41], [42] was utilized to obtain the functional enrichments within the 184 genes conserved at amino acid level, yet having significant epigenetic differences at their promoter. The data set containing 7,252 human∶chimpanzee 1∶1 orthologs was used as a background.

## Supporting Information

Figure S1 Effect of sequence mismatches on probe performance. Difference in mean β-values between human and chimpanzee (A), gorilla (B) and orangutan (C) for each category of probes. Probes with mismatches located in the last 5 bp at the 3′ end, C>T transitions of the CpG site being measured, or ≥3 bp of mismatch showed an excess of variation compared to probes with ≤2 mismatches, and were removed from further analysis.
(PDF)

Figure S2 Effect of sequence mismatches on probe performance. Distribution of differences in mean β-value between human and (A) chimpanzee, (B) gorilla and (C) orangutan. Dotted lines represent the probes used in this study (perfect match and 1–2 mismatches).
(PDF)

Figure S3 Neighbor-joining tree based on 114,739 autosomal CpGs measured in all individuals and species. Bootstrap values (1,000 permutations) are shown for each node.
(PDF)

Figure S4 (A) Density plot showing the distribution of methylation levels of CpG sites in males and females. (B) The *HTR2C* gene on the X chromosome shows a relative reduction in promoter methylation specifically in human females compared to other great ape species. Probes upstream of the TSS of *HTR2C* show similar patterns of methylation in both male and female humans. The same sites show significantly increased methylation in females versus males in all other primate species tested. These observations suggest evolutionary changes in the X chromosome inactivation status of *HTR2C* specifically in humans compared to other primates.
(PDF)

Figure S5 Comparison of alterations in promoter methylation with frequency of amino-acid changes and the relative rate of coding to non-coding variation within genes (K_A_/K_I_) between human and chimpanzee in two data sets. (A) Probes with perfect match to the chimpanzee genome. Differentially methylated genes: n = 334, genes without significant changes in methylation: n = 5,655. (B) Probes with 1 or 2 mismatches in the first 45 bp in the chimpanzee genome. Differentially methylated genes: n = 247, genes without significant changes in methylation: n = 4,840.
(PDF)

Figure S6 (A) β-value distribution of the 114,739 sites shared among the five species before and after BMIQ. (Infinium type I probe, n = 32,216. Infinium type II probes, n = 82,523). (B) β-value distribution of the 291,553 sites shared among human and chimpanzee before and after BMIQ, (Infinium type I probe, n = 83,528. Infinium type II probes, n = 208,025).
(PDF)

Table S1 Sample information. NA: Information not available for these samples.
(XLS)

Table S2 10,404 CpG sites showing species-specific differential methylation.
(XLS)

Table S3 815 genes associated with species-specific methylation changes.
(XLS)

Table S4 Results of GREAT analysis showing GO terms significantly associated with methylation changes in human, *Pan* species, and *Pongo* species. All GO terms shown have p<0.05 (5% FDR).
(XLS)

Table S5 CpG sites on the X chromosome showing gender-specific methylation changes.
(XLS)

Table S6 184 genes that show perfect conservation of amino acid sequence between human and chimpanzee, but which have significant epigenetic changes within their promoter regions.
(XLS)

Table S7 184 genes that show perfect conservation of amino acid sequence between human and chimpanzee, but which have significant epigenetic changes within their promoter regions.
(XLSX)

Text S1 Supplementary methods.
(DOCX)
