# Supplementary figures and images for: Dynamics of DNA Methylation in Recent Human and Great Ape Evolution
Source: PLoS Genet. 2013 Sep 5;9(9):e1003763. doi: 10.1371/journal.pgen.1003763 (PMC3764194; doi:10.1371/journal.pgen.1003763)

Figure S1:

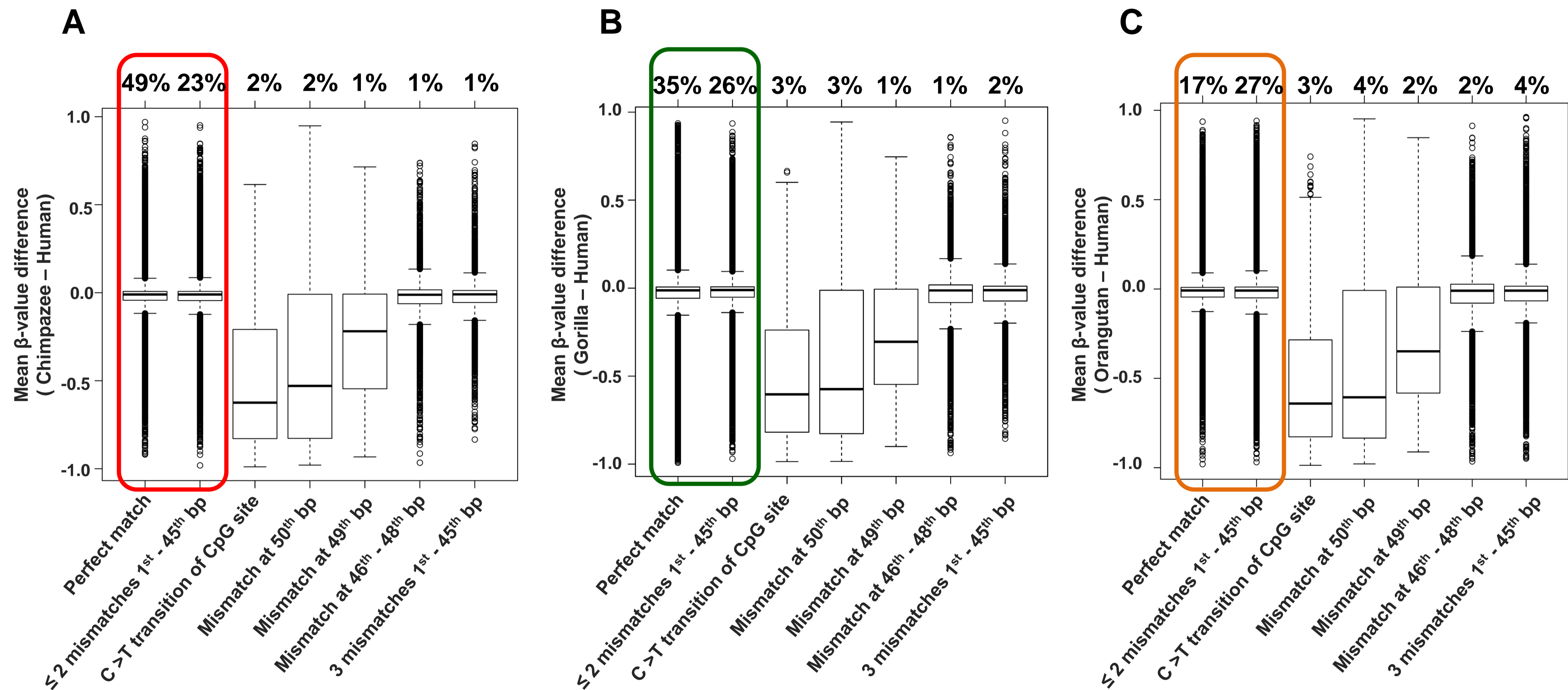

Supplement: Figure S1 — Effect of sequence mismatches on probe performance. Difference in mean β-values between human and chimpanzee (A), gorilla (B) and orangutan (C) for each category of probes. Probes with mismatches located in the last 5 bp at the 3′ end, C>T transitions of the CpG site being measured, or ≥3 bp of mismatch showed an excess of variation compared to probes with ≤2 mismatches, and were removed from further analysis. (PDF) [file pgen.1003763.s001.pdf]

Figure S3:

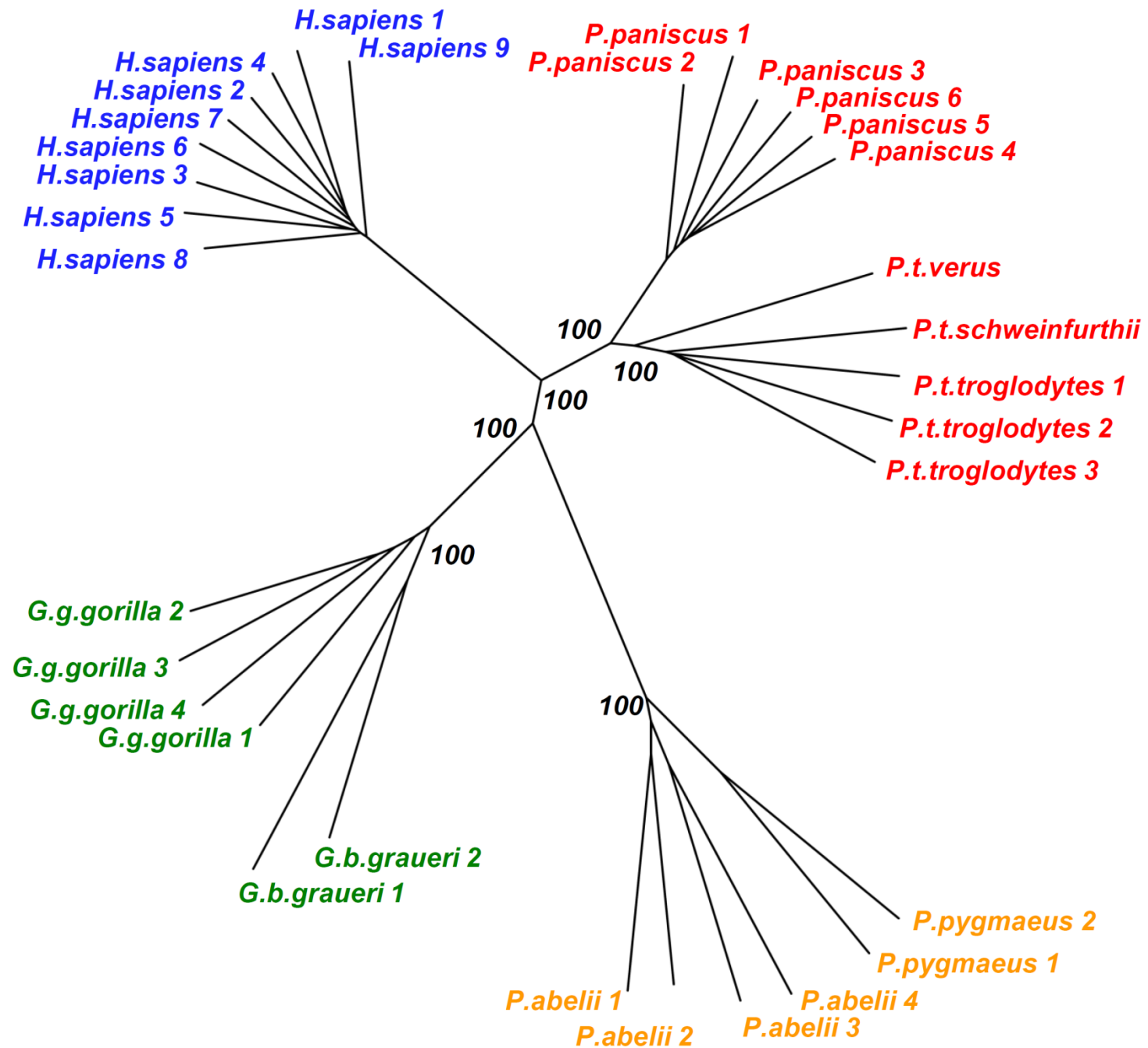

Supplement: Figure S3 — Neighbor-joining tree based on 114,739 autosomal CpGs measured in all individuals and species. Bootstrap values (1,000 permutations) are shown for each node. (PDF) [file pgen.1003763.s003.pdf]

Figure S4:

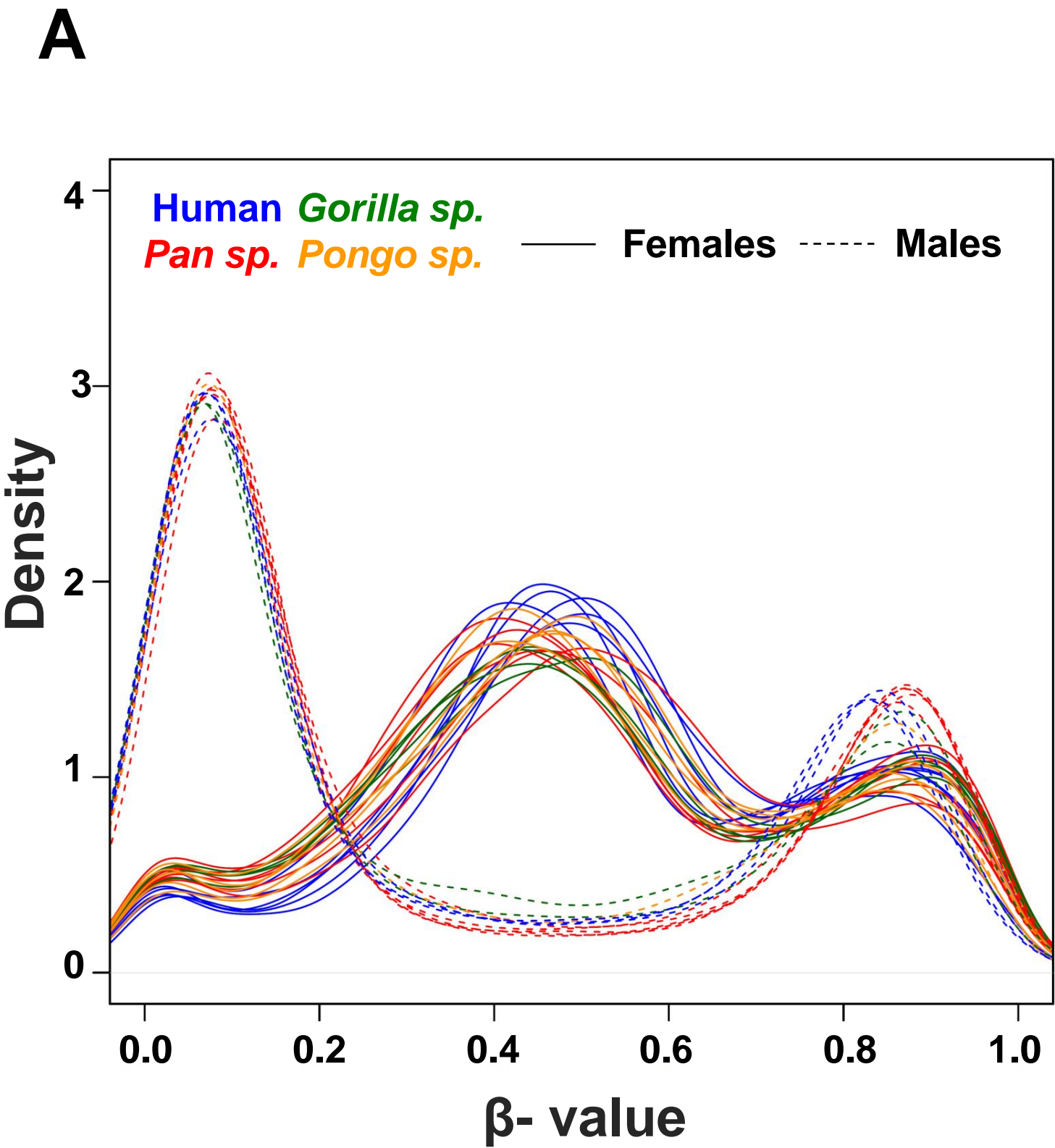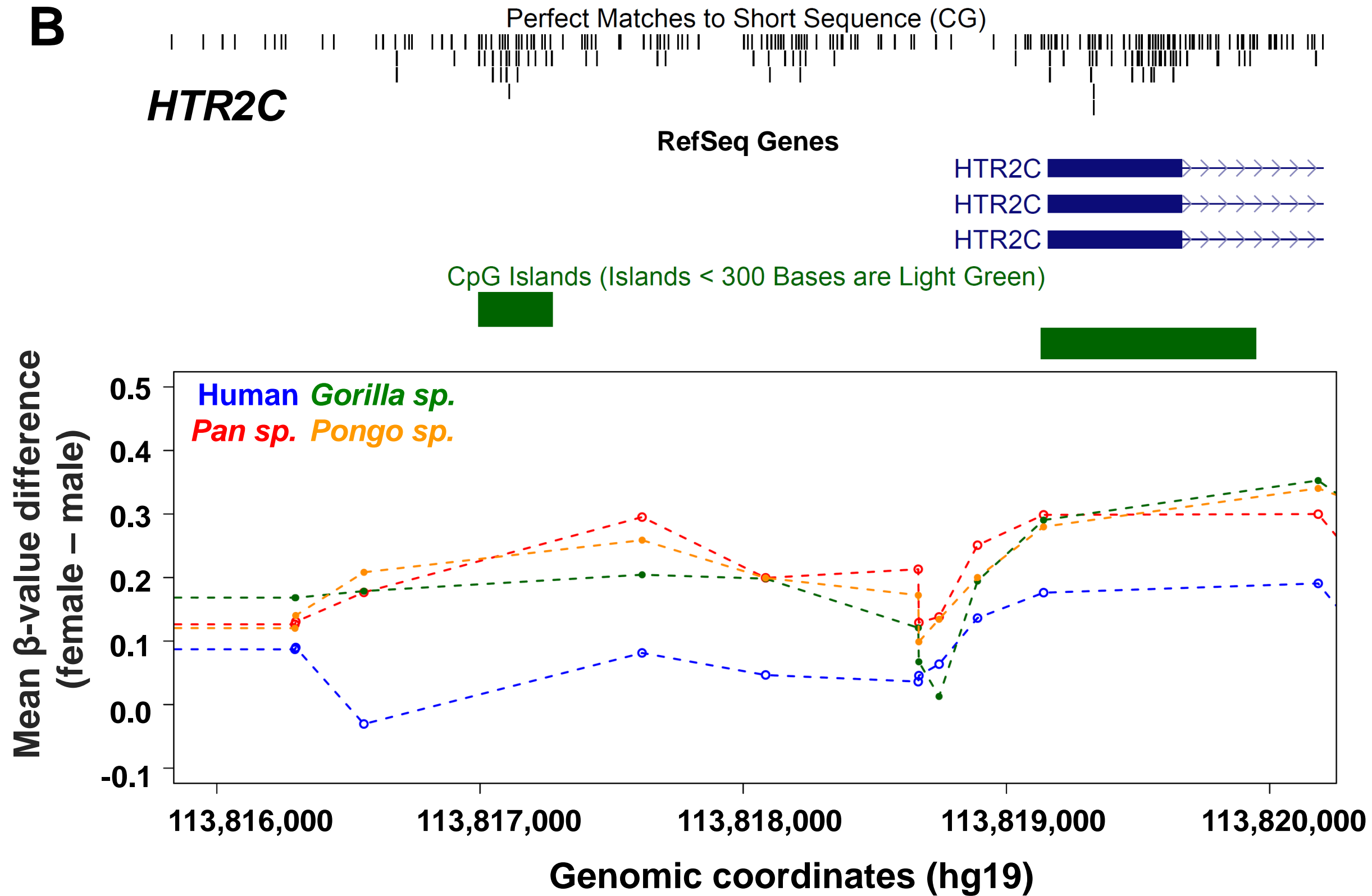

Supplement: Figure S4 — (A) Density plot showing the distribution of methylation levels of CpG sites in males and females. (B) The HTR2C gene on the X chromosome shows a relative reduction in promoter methylation specifically in human females compared to other great ape species. Probes upstream of the TSS of HTR2C show similar patterns of methylation in both male and female humans. The same sites show significantly increased methylation in females versus males in all other primate species tested. These observations suggest evolutionary changes in the X chromosome inactivation status of HTR2C specifically in humans compared to other primates. (PDF) [file pgen.1003763.s004.pdf]

Figure S5:

**A** Perfect match probes

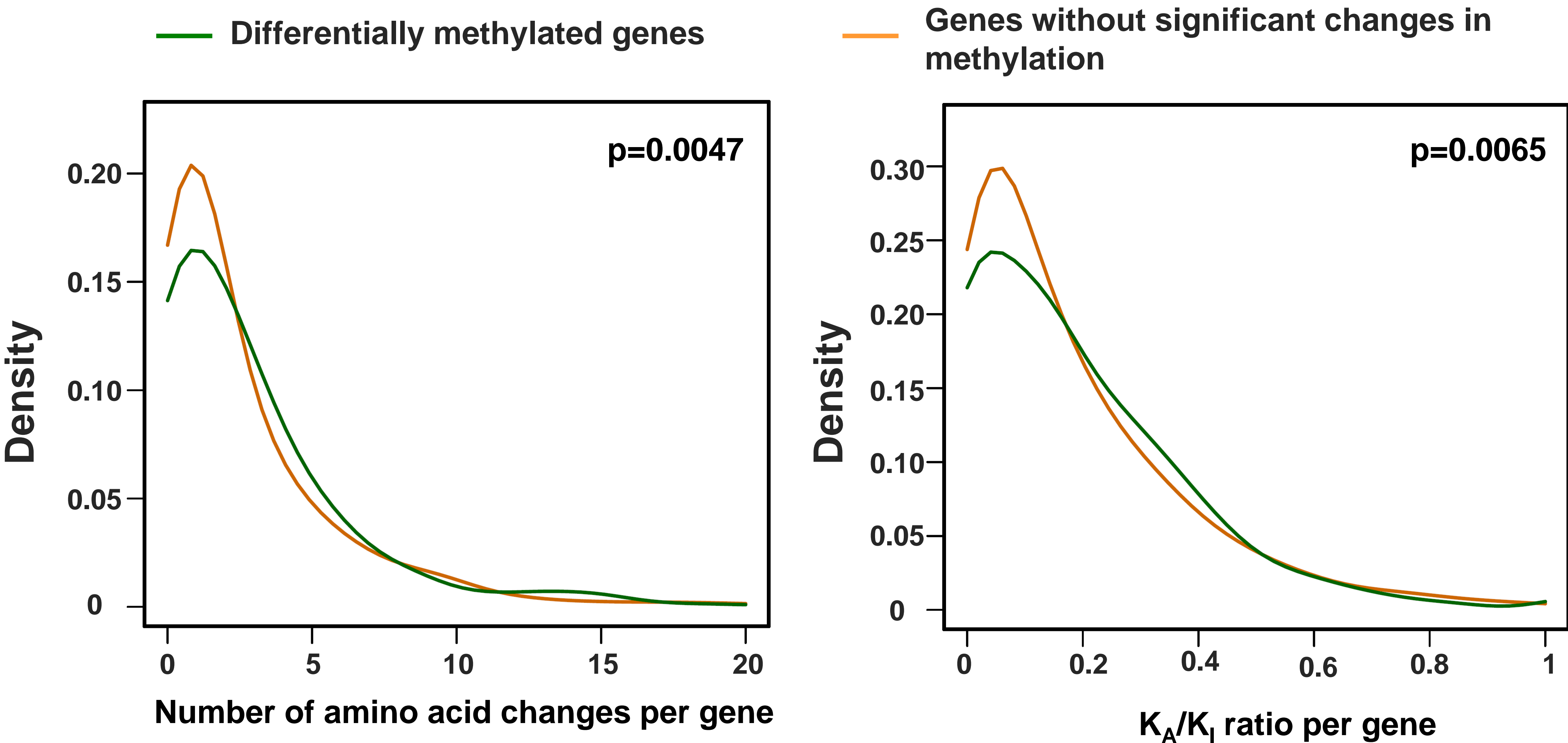

**B** Probes with 1-2 mismatches in the 1<sup>st</sup> – 45<sup>th</sup> bp

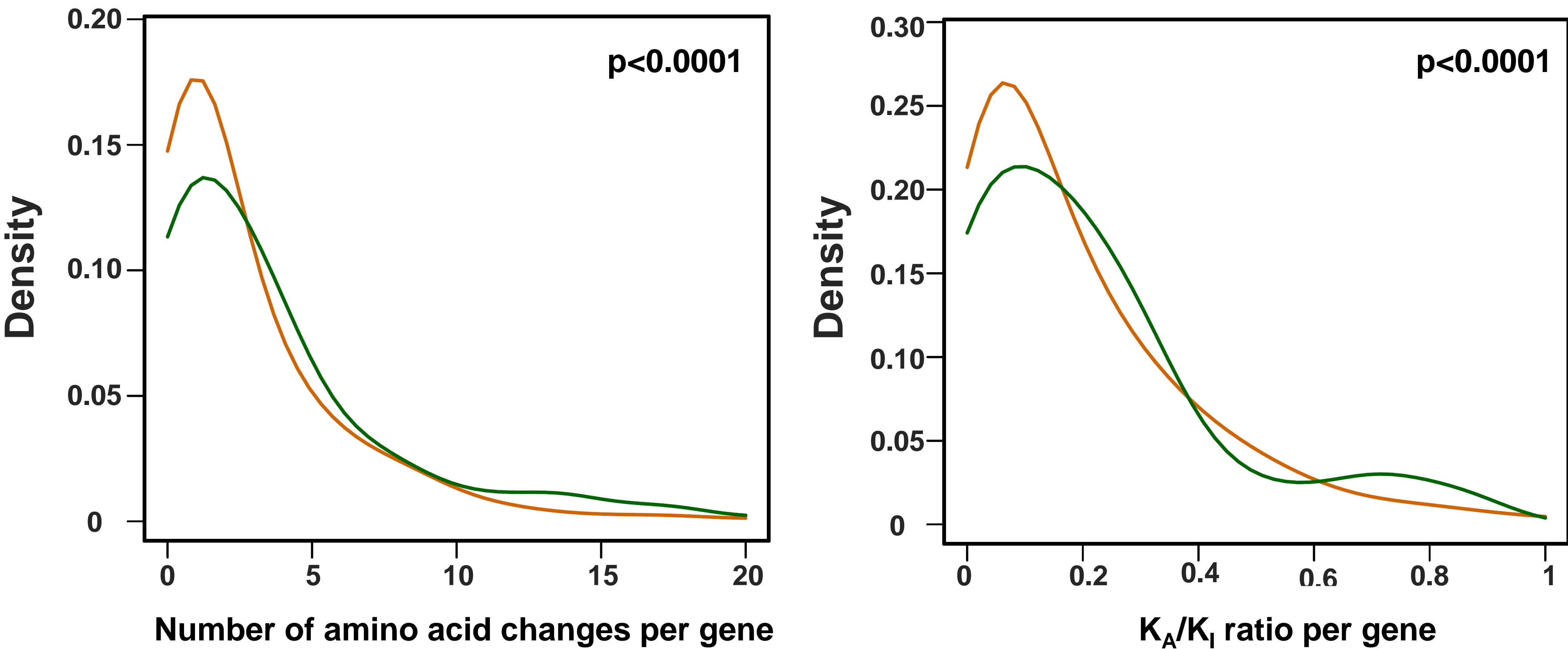

Supplement: Figure S5 — Comparison of alterations in promoter methylation with frequency of amino-acid changes and the relative rate of coding to non-coding variation within genes (KA/KI) between human and chimpanzee in two data sets. (A) Probes with perfect match to the chimpanzee genome. Differentially methylated genes: n = 334, genes without significant changes in methylation: n = 5,655. (B) Probes with 1 or 2 mismatches in the first 45 bp in the chimpanzee genome. Differentially methylated genes: n = 247, genes without significant changes in methylation: n = 4,840. (PDF) [file pgen.1003763.s005.pdf]

**Figure S6:**

**A**

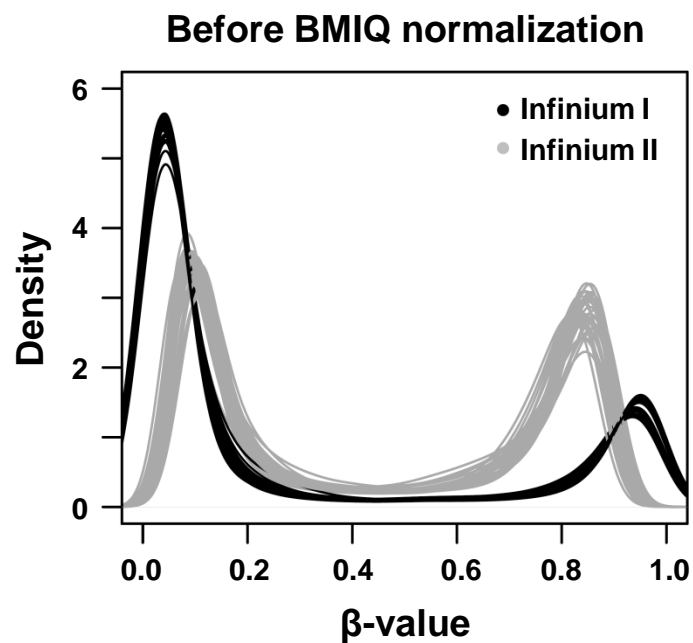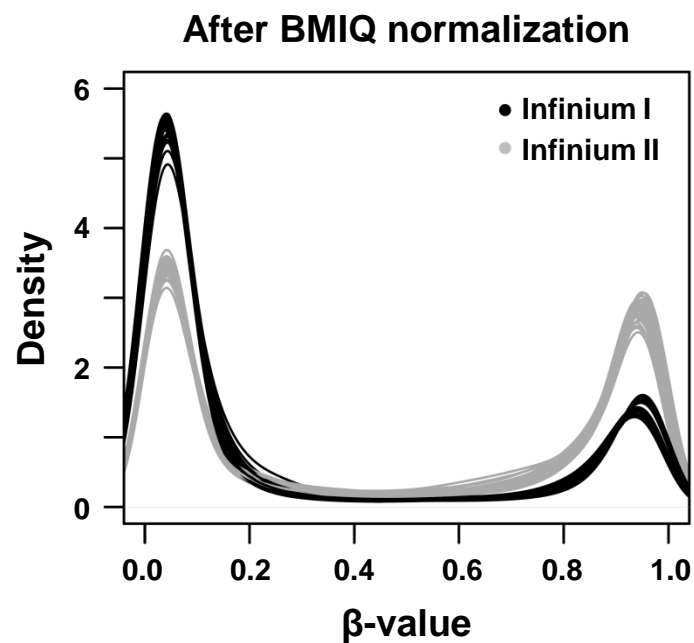

**B**

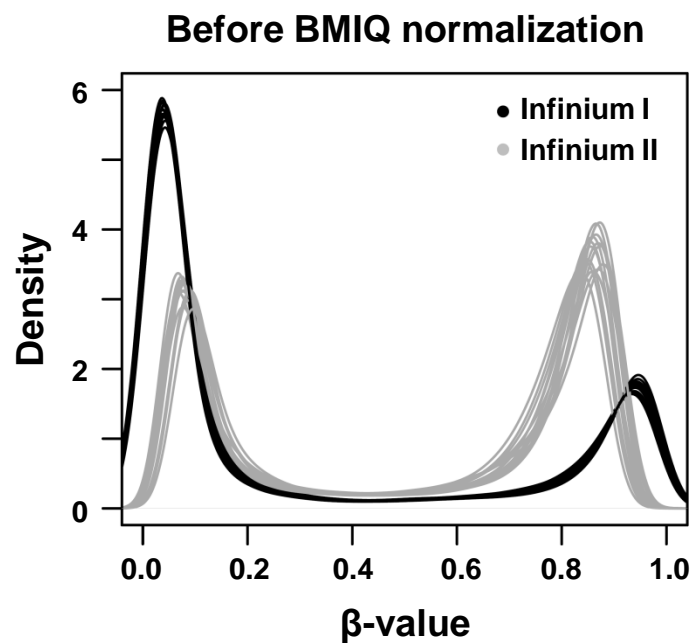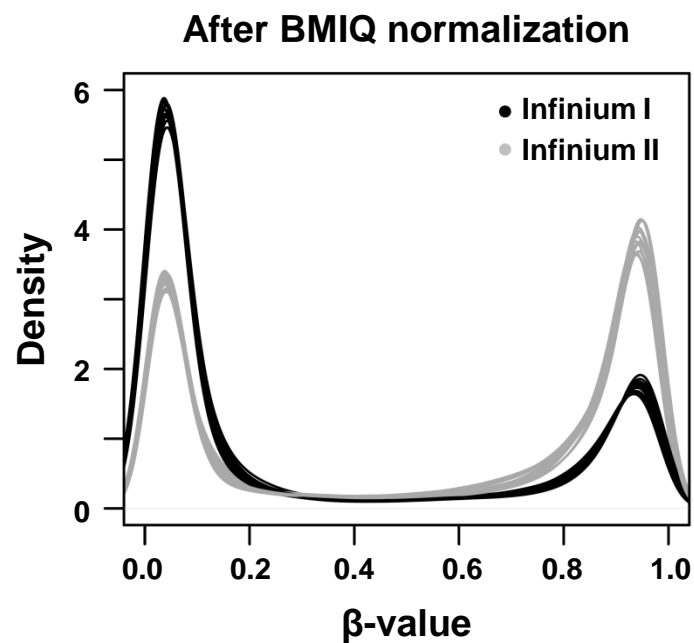

Supplement: Figure S6 — (A) β-value distribution of the 114,739 sites shared among the five species before and after BMIQ. (Infinium type I probe, n = 32,216. Infinium type II probes, n = 82,523). (B) β-value distribution of the 291,553 sites shared among human and chimpanzee before and after BMIQ, (Infinium type I probe, n = 83,528. Infinium type II probes, n = 208,025). (PDF) [file pgen.1003763.s006.pdf]
