# Supplementary material for: Dynamics of DNA Methylation in Recent Human and Great Ape Evolution
Source: PLoS Genet. 2013 Sep 5;9(9):e1003763. doi: 10.1371/journal.pgen.1003763 (PMC3764194; doi:10.1371/journal.pgen.1003763)
Supplement: Figure S2 — Effect of sequence mismatches on probe performance. Distribution of differences in mean β-value between human and (A) chimpanzee, (B) gorilla and (C) orangutan. Dotted lines represent the probes used in this study (perfect match and 1–2 mismatches). (PDF) [file pgen.1003763.s002.pdf]

Figure S2:

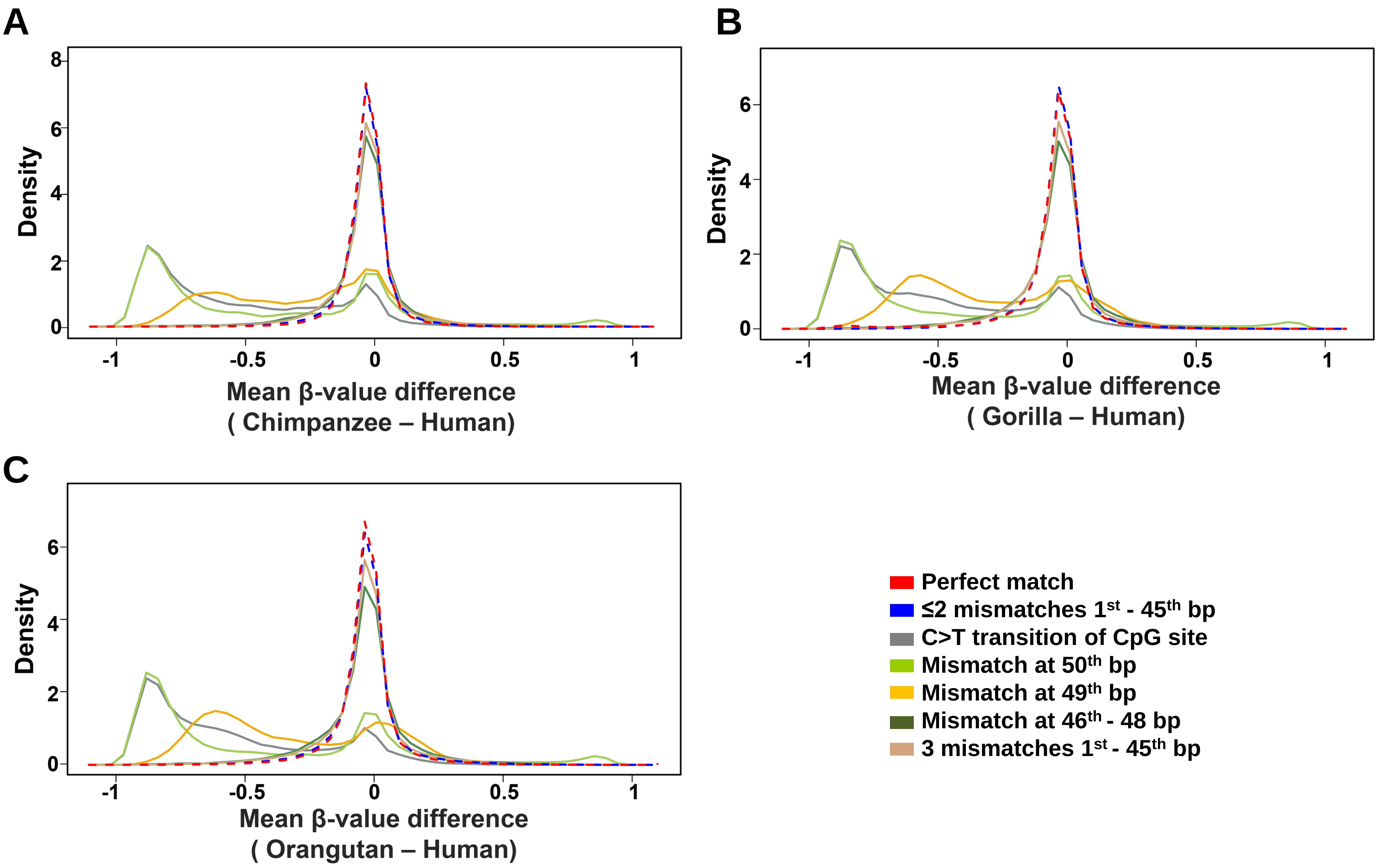

A
B
C
Density
Mean β-value difference
(Chimpanzee – Human)
(Gorilla – Human)
(Orangutan – Human)
Perfect match
≤2 mismatches 1st - 45th bp
C>T transition of CpG site
Mismatch at 50th bp
Mismatch at 49th bp
Mismatch at 46th - 48 bp
3 mismatches 1st - 45th bp
