# Supplementary material for: Dynamics of DNA Methylation in Recent Human and Great Ape Evolution
Source: PLoS Genet. 2013 Sep 5;9(9):e1003763. doi: 10.1371/journal.pgen.1003763 (PMC3764194; doi:10.1371/journal.pgen.1003763)
Supplement: Text S1 — Supplementary methods. (DOCX) [file pgen.1003763.s014.docx]

**Text S1.**

We tested whether the rate of accumulation of methylation changes is uniform or different in the interrogated branches. To do so, we followed a previous approach [48] and built a Likelihood Ratio Test (LRT) framework that tests whether four independent rates of acquisition of methylation explain our observations significantly better than a single one. This test ignores homoplasy and assumes a simple Poisson rate of methylation.

As a measure of methylation changes we used two different units: a) Genus-specific sites without differences in the other three genera and b) clusters of these sites into regions with at least two close differentially methylated CpGs (<1kb interval) and overlapped with RefSeq genes (-1500bp from TSS to 3’UTR). As a unit of time/branch length, we used the split time of the corresponding branches (in Myrs) [26]. The following table indicates the units of methylation changes and time in the branches being tested:

|  | **Individual CpG sites** | **Genes** | **Split Time** |
| --- | --- | --- | --- |
| **Human** | 1,286 | 86 | 4.5 |
| ***Pan sp.*** | 655 | 33 | 3.5 |
| ***Gorilla sp.*** | 651 | 26 | 5.03 |
| ***Pongo sp.*** | 3,902 | 256 | 12 |

To perform the test, we first obtained maximum-likelihood estimates for two different models. The simplest one assumes a single rate of accumulation of changes in methylation everywhere and the other one assumes that every branch has its own rate. Afterwards, we performed a Likelihood Ratio Test between the two models; we use 3 degrees of freedom since the second model has three more parameters.

The results of the two tests that can be performed with the two units of methylation changes (CpG sites and genes) are shown below.

| **Accumulation of CpG sites changes/ Myrs Since Split** | | | |
| --- | --- | --- | --- |
|  | **Model 1**  **(all identical rate)** | **Model 2**  **(four different rates)** | **LRT**  **p-value** |
| **One vs. Four rates** | λ = 259,45 MetRs/Myr | λ_Hum_ = 285,78  λ_Pan_ = 187,14  λ_GGO_ = 129,42  λ_Pon_ = 325,17 | <10^-10^ |
|  |  |  |  |

| **Accumulation of Gene methylation changes/ Myrs Since Split** | | | |
| --- | --- | --- | --- |
|  | **Model 1**  **(all identical rate)** | **Model 2**  **(four different rates)** | **LRT**  **p-value** |
| **One vs. Four rates** | λ = 16,02 MetRs/Myr | λ_Hum_ = 19.11  λ_Pan_ = 9.42  λ_GGO_ = 5.16  λ_Pon_ = 21.33 | <10^-10^ |

In both tests, four rates explain our observations better than a single rate.
